# Concordance between self-reported and measured HIV and hepatitis C virus infection status among people who inject drugs in Germany

**DOI:** 10.1186/s41124-016-0016-6

**Published:** 2016-09-01

**Authors:** Stine Nielsen, Martyna Gassowski, Benjamin Wenz, Norbert Bannert, Claus-Thomas Bock, Claudia Kücherer, R. Stefan Ross, Viviane Bremer, Ulrich Marcus, Ruth Zimmermann, the DRUCK study group, Vikas Bapat, Johannes Bombeck, Kerstin Dettmer, Andreas Hecht, Werner Heinz, Christiane Kerres, Jürgen Klee, Astrid Leicht, Sylke Lein, Bärbel Marrziniak, Olaf Ostermann, Norbert Scherbaum, Dirk Schäffer, Ina Stein, Lutz Wiederanders

**Affiliations:** 1 Department for Infectious Disease Epidemiology, Division for HIV/AIDS, STI and Blood-borne Infections, Robert Koch Institute, Berlin, Germany; 2 Charité University Medicine, Berlin, Germany; 3 Department for Infectious Diseases, Division for HIV and other Retroviruses, Robert Koch Institute, Berlin, Germany; 4 Department for Infectious Diseases, Division for Viral Gastroenteritis and Hepatitis Pathogens and Enteroviruses, Robert Koch Institute, Berlin, Germany; 5 Institute of Virology, National Reference Centre for Hepatitis C, University Hospital Essen, University of Duisburg-Essen, Essen, Germany

**Keywords:** People who inject drugs, Germany, Hepatitis C, HIV, Testing, Knowledge, Self-report, Validity, Undiagnosed, Respondent driven sampling

## Abstract

**Background:**

People who inject drugs (PWID) are disproportionately affected by both HIV and hepatitis C infection (HCV). Awareness of infection status is essential to ensure linkage to appropriate healthcare for those infected, who need treatment and regular follow-up, as well as for uninfected individuals, who need access to targeted testing and counselling services. In this paper we compare self-reported HIV and HCV status with serological markers of infection among PWID recruited through respondent driven sampling.

**Methods:**

From 2011 through 2014, biological and behavioural data was collected from 2,077 PWID in Germany. Dried blood spots from capillary blood samples were collected and screened for HCV antibodies, HCV RNA and HIV-1/-2 antibodies. HIV reactive samples were confirmed by Western blot.

**Results:**

Laboratory testing revealed that 5 % were infected with HIV and 81 % were aware of being infected. Chronic HCV infection was detected in 41 % of the participants, 2 % had an acute HCV infection, 22 % had a cleared infection, and 34 % were unexposed to HCV. The concordance between self-reported and measured HCV status was lower than for HIV, with 73 % of those with chronic HCV infection being aware of their infection.

**Conclusions:**

We found a relatively high awareness of HIV and HCV infection status among PWID. Nevertheless, access to appropriate testing, counselling and care services targeted to the needs of PWID should be further improved, particularly concerning HCV.

**Trial registration:**

Ethical approval was received from the ethics committee at the medical university of Charité, Berlin, Germany in May 2011 and with an amendment approved retrospectively on 19/11/2012 (No EA4/036/11). The German Federal Commissioner for Data Protection and Freedom of Information approved the study protocol retrospectively on 29/11/2012 (III-401/008#0035).

## Background

Accurate knowledge of infection status is important as it gives infected individuals the opportunity to seek appropriate healthcare and may encourage people to engage in preventive behaviours, which can protect themselves and others from infections. People who inject drugs (PWID) have a high risk and burden of both HIV and Hepatitis C infection (HCV) [1–4]. Determining HIV infection status is relatively straightforward since there is no clearance or cure. In contrast, screening for HCV antibodies (anti-HCV) will identify if a person has ever been in contact with the virus, but this person may have cleared the infection either with treatment or spontaneously, or the person may have a chronic HCV infection, characterised by being both anti-HCV and HCV RNA positive. A person who has cleared the infection can later be re-infected with HCV. Since the acquisition of both HIV and HCV is often asymptomatic or the occurrence of non-specific symptoms may be attributed to other problems, and since serious sequelae may take several decades to develop, people infected with these two viruses may remain unaware of being infected for a long period of time. Annual routine unlinked anonymous monitoring (UAM) of HIV and hepatitis among PWID in England, Wales and Northern Ireland has shown that the proportion of infected PWID unaware of their infection varied between 4 %-15 % for HIV and 45 %-53 % for HCV in the period 2010–2014 [5]. In a recent systematic review including 11 studies from five EU countries the proportion of undiagnosed HCV infections among PWID varied between 24 %-76 % (IQR: 38 %-64 % and median: 49 %) [4]. Several previous studies only looked at anti-HCV status and did not include HCV RNA status, which is needed to assess current infection status [4–6].

With new, highly effective and well tolerable HCV therapy options being available as well as the potential of HIV-treatment as prevention also among PWID [7], it is of growing importance to increase awareness of infection status.

In order to assess the level of awareness of infection status among PWID in Germany, we used data from a recent, cross-sectional bio-behavioural survey of this population to compare self-reported HIV and HCV status with serological markers of infection.

## Methods

The DRUCK-study collected biological and behavioural data from 2,077 PWID in eight large German cities in the years 2011–2014 [8]. The respondents were recruited using respondent driven sampling. Inclusion criteria were a minimum age of 16 years, having injected drugs in the given study city in the last 12 months, and providing informed consent for study participation. All participants went through a questionnaire-assisted interview and provided a capillary blood sample, collected as dried blood spots (DBS). Samples and questionnaires were marked with the same unique identifier. The study was piloted in two cities and then implemented in the remaining six. Before starting the data collection we trained interviewers to increase their understanding of HIV and HCV, and laboratory staff for collection of DBS and handling and shipping of the samples in each of the study cities to ensure the comparability of results. All samples were screened for anti-HIV-1/-2 by EIA, and reactive samples were confirmed by Western blot. In six of the eight study cities, all samples were screened for both anti-HCV by EIA and analysed for the presence of HCV-RNA using nested RT-PCR. Anti-HCV positive samples were confirmed by immunoblot. In the two pilot cities all samples were screened for anti-HCV by EIA and RT-PCR was performed on all anti-HCV positive samples and on anti-HCV negative samples if test results did not correspond to self-reported results. The test specificity was 100 % for all three markers: anti-HIV, anti-HCV and HCV RNA. The same was true for the sensitivity for HIV and HCV RNA, whereas the sensitivity for anti-HCV was 97.8 % [9]. Further information about study design, data collection and laboratory methods etc. has been published elsewhere [8, 9].

### Defining self-reported HIV status

The self-reported HIV status was determined using two questions: if the participant had ever been tested for HIV and if yes, what the result of their latest test was. Based on the answers participants were categorised as HIV negative, HIV positive or never tested. Participant’s self-reported HIV status were categorised as unclear when they were not sure if they had ever been tested or if they did not know their last test result. Participants who reported having been diagnosed with HIV were asked about month and year of their first positive HIV test in order to calculate how long they had been aware of being infected.

### Defining measured HIV status

We defined samples as HIV positive if testing positive for anti-HIV with EIA and being confirmed by Western blot. EIA-reactive samples with indeterminate immunoblot pattern were excluded from this analysis. Anti-HIV negative samples were determined as HIV negative.

### Defining self-reported HCV status

To determine the self-reported HCV status several questions were used. Participants were asked if they had ever been tested for anti-HCV. Those who had not were categorised as never tested. The participants who reported testing for HCV were further asked if they had ever received a positive anti-HCV test result. Those who had not were categorised as uninfected. Participants reporting having ever received a positive anti-HCV test result were further asked if they had ever been successfully treated or had cleared the infection spontaneously. Those who responded no to both questions were categorised as infected, while those who responded yes to either of the questions were defined as previously infected. As for HIV, participants who were either not sure if they had ever been tested or did not know their last test result, were categorised as unclear.

### Defining measured HCV status

We defined chronic HCV infection as testing anti-HCV and RNA positive, acute infection (HCV infection acquired within the last 4–6 weeks) as anti-HCV negative, but RNA positive, cleared infection as anti-HCV positive and HCV RNA negative, and unexposed as testing anti-HCV and RNA negative.

An example of the questionnaire used to guide the interviews can be found online [10].

## Results

In total, 2,077 PWID from eight German cities were included in the study, with the proportion of female participants ranging between 19 %-35 % in the respective cities, and a median age ranging between 29–41 years.

### HIV status

The laboratory testing revealed 100 participants (4.8 %) to be positive for HIV, and 1976 (95.2 %) to be negative. One sample had a reactive EIA result but an indeterminate immunoblot, and was thus excluded from the analysis. Of the 100 HIV positive cases, 81 % were aware of their infection while 16 % reported their last HIV test to be negative. One HIV positive participant reported no previous testing.

Among the participants testing negative for HIV, 90 % reported a negative test result at last test, while 7 % reported never having had an HIV test. Of the HIV negative participants, six individuals (0.3 %) reported having received a positive HIV-test result. Two HIV positive and one HIV negative participant declined to answer the question on HIV-status. The concordance of the self-reported HIV status and laboratory test results is displayed in Table 1.

**Table 1:** Concordance of self-reported and measured HIV status, *n*=2076 (excluding one sample with indeterminate HIV status)

	HIV laboratory test results	
Self-reported status	HIV negative (AB-)	HIV positive (AB+)
Concordant	1784 (90 %)	81 (81 %)
Discordant	6 (0,3 %)	16 (16 %)
Never tested	133 (7 %)	1 (1 %)
Unclear	52 (3 %)	0 (0 %)
Answer declined	1 (0,1 %)	2 (2 %)
Total	1976	100

Unclear means not sure if tested or did not get last test result.

*AB* antibodies

Among the 81 self-reported HIV infected, 5 % reported receiving their diagnosis in the last year, 17 % 1–5 years ago, 22 % 6–10 years ago and 47 % more than 10 years ago. Seven cases (9 %) did not provide information on time of their HIV diagnosis.

### HCV status

The laboratory tests found 716 (34 %) participants to be unexposed to HCV, 857 (41 %) participants to have a chronic HCV infection and 457 (22 %) participants with a cleared HCV infection. In 47 (2 %) participants HCV-RNA but no anti-HCV was detected, indicating an acute infection. As the participants were only asked about anti-HCV test results, this group was excluded from the analysis.

The concordance between the self-reported HCV status and the laboratory test results was 47 % among those unexposed, while 27 % reported an HCV status discordant to the laboratory findings (Table 2). Of these, 56 % reported a current HCV infection and 44 % a previous one (Table 3). Of all unexposed individuals, 16 % reported never to have had a test for HCV. In the group with confirmed, chronic HCV infections, 73 % reported a status concordant to the laboratory test results, whereas 19 % reported a differing HCV status. In 37 % of these discordant cases, participants reported to be uninfected and in 63 % a cleared infection. Among participants positive only for anti-HCV but not HCV-RNA, i.e., with a cleared infection, 38 % correctly reported to have cleared the infection. Of those reporting a discordant status, 89 % reported to be currently infected and 11 % reported an uninfected status.

**Table 2:** Concordance of self-reported and measured HCV status, *n*=2030 (excluding cases with acute infection)

	HCV laboratory test results		
Self-reported status	Unexposed (AB-, RNA-)	Chronic infection (AB+, RNA+)	Cleared infection (AB+, RNA-)
Concordant	339 (47 %)	622 (73 %)	174 (38 %)
Discordant	194 (27 %)	163 (19 %)	254 (56 %)
Never tested	113 (16 %)	37 (4 %)	15 (3 %)
Unclear	69 (10 %)	35 (4 %)	14 (3 %)
Answer declined	1 (0,1 %)	0 (0 %)	0 (0 %)
Total	716	857	457

Unclear means not sure if tested or did not get last test result

*AB* antibodies

**Table 3:** Discordance of self-reported and measured HCV status, *n*=611

	HCV laboratory test results		
Self-reported status	Unexposed (AB-, RNA-)	Chronic infection (AB+, RNA+)	Cleared infection (AB+, RNA-)
Uninfected		61 (37 %)	29 (11 %)
Infected	109 (56 %)		225 (89 %)
Previously infected (cleared infection)	85 (44 %)	102 (63 %)	
Total	194	163	254

*AB* antibodies

## Discussion

In our study population of PWID, the concordance of self-reported and measured HIV status was relatively high. The proportion of HIV positive participants aware of their infection was 81 %. These data compare well with the data from both the UAM in England, Wales and northern Ireland from 2010–2014 where 85-96 % of HIV positive PWID were aware of their HIV infection [5] and the latest HIV modelling data from Germany, where it was estimated that 89 % (81-93 %) of all HIV infected PWID living in Germany in 2014 had received an HIV diagnosis [11]. According to the same HIV modelling data for Germany, the proportion of HIV infected individuals who are aware of their HIV status is higher among PWID compared to other groups such as men who have sex with men (MSM) (82 % (79-85 %)) and non-injecting heterosexuals (74 % (66-80 %)). This might partly be explained by the fact that the majority of HIV infections among PWID in Germany were acquired in the 1980ies and 90ies, which is also seen by the high proportion of known HIV infections being diagnosed more than 10 years ago. This means that the majority of HIV infected PWID have had many years to get a diagnosis and begin therapy. Increasing treatment rates among PWID may have a relatively large impact on reducing potential sources of HIV transmission within this population [7]. A low rate of newly acquired HIV infections is also indicated by the fact that <1 % of PWID not yet tested for HIV were found to be anti-HIV positive in our study. The one sample with a reactive AB test and indeterminate immunoblot could be a recent infection in the stage of seroconversion. We tried to receive a second blood sample from this participant to repeat the testing, but the person did not show up in the drug service again.

The finding that 17 % of those positive for HIV reported a negative HIV status or to never have been tested, underlines the undiminished importance of ensuring access to targeted HIV testing and counselling services for PWID in Germany, e.g., in low threshold settings.

For the disconcerting finding of six self-reported infections in participants without measurable anti-HIV several possible explanations exist. One possibility is the failure to detect antibodies in excessively diluted samples if the original amount of capillary blood was inadequately small, resulting in a higher dilution of the antibodies as compared to the standardized and validated protocol. Further we cannot rule out the communication of false positive test results to study participants, in particular if reactive screening test results were not confirmed, which can happen if respondents were not linked into care. Processing mistakes during sample collection or during testing of DBS, or the disappearance of HIV antibodies if treatment has been started early and viral load has remained suppressed continuously are further, though in our view less likely possibilities.

The results of the HCV testing revealed that 34 % were unexposed to HCV, 2 % had an acute HCV infection, 41 % of participants had a chronic infection and 22 % had a cleared infection. It is possible that a few acute HCV infections might have been missed in the two first pilot cities where PCR was only done for anti-HCV negative respondents with a self-reported HCV diagnosis. The concordance between self-reported and actual HCV status was much lower than for HIV. Concordance was highest (73 %) among those with a chronic infection, 47 % among those unexposed to HCV and just 38 % among those with a cleared infection.

The somewhat surprising finding of 27 % of those with no markers of HCV infection reporting to be infected (chronic or cleared HCV), might partly be explained by confusion about the different types of hepatitis, e.g., participants may previously have received a positive test result for hepatitis B. Another explanation could be the failure to detect antibodies e.g., due to excessive dilution, as described above for HIV or a false negative anti-HCV test result which is not unlikely given the estimated sensitivity of 97.8 %.

Of the participants with a chronic HCV infection, 27 % falsely believed to be uninfected, to have cleared the infection or had never been tested or were not sure of their test result. This rate is much lower than what is reported from the UAM in England, Wales and northern Ireland where 45-53 % of anti-HCV positive were unaware of their HCV infection in the years 2010–2014 [5]. Also the recent systematic review on HCV in PWID in Europe found higher rates of undiagnosed HCV: 24-76 % (IQR: 38-64 % and median: 49 %) [4]. An Australian study of 352 active injectors under the age of 30, found rates of concordance similar to our study: 68 % among those with a chronic infection and 46 % among those with a cleared HCV infection [12]. Also data from a French study of HCV among PWID found that only 22 % of PWID were unaware of their HCV infection [13, 14]. A recently published study from Spain presents data on the proportion of undiagnosed HCV infection among PWID stratified by migration status and duration of injecting. This study reports rates of undiagnosed HCV from 15 % among Spanish long-term injectors up to 57 % among migrant new injectors [6]. All these data suggest that awareness of HCV infection status among PWID in Germany is relatively high. This is also true when comparing with data for the general population in Europe, where between 40 % and 80 % of people with chronic hepatitis are believed to be unaware of their infection [15]. However, the persons indicating a true positive anti-HCV test result often do not necessarily also know their PCR test result. In times of effective treatment options for HCV this will become increasingly important.

In our study, the majority (63 %) of those with a chronic HCV infection with a discordant self-reported status were aware of having been exposed to the virus but believed to have cleared the infection. These participants might have simply assumed to be healed (we did not collect data on whether the reported clearance had been laboratory confirmed) or they might indeed have cleared the virus but later become re-infected. These results show the need of special screening efforts of PWID who have once cleared their HCV infection, as re-infections can only be diagnosed by detecting the viral RNA.

From a public health point of view, the most undesirable discordant status is being infected and either not being aware of being infected or believing to be uninfected. This discrepancy between perception and reality limits the access to appropriate health care and may increase the risk of unknowingly transmitting infections. However, the evidence about the association between knowledge of HCV status and risk behaviours in PWID is conflicting. Some longitudinal studies have observed a reduction in risky injecting drug use following notification of HCV-positive status [16, 17], while other studies found either no reduction or even increased risky injection behaviours among PWID receiving a diagnosis of HCV infection [18, 19]. This means that believing to be infected, while actually being uninfected may turn out to become a self-fulfilling prophecy. As mentioned in the results, it was common among both unexposed participants, as well as participants with a cleared infection, to wrongly believe they were currently infected. It might be that those who have been at risk assume they are infected and/or do not believe their test results.

Studying HCV infection status and HCV test status is complicated and not only our study participants, but in our experience also both non-medical and medical staff often had difficulties in distinguishing between the two HCV tests (AB and RNA) and in interpreting the combination of the two test results. Our findings, as are those from other, similar studies, are limited by the lack of clarity regarding HCV test and infection status among both interviewers and respondents. Several qualitative studies among PWID have shown that confusion and uncertainty regarding the meaning of a positive HCV test and HCV risk exist in this group and that HCV is often perceived as an almost inevitable consequence of drug injecting [20–22].

Our data is collected from eight large cities in Germany, but is not likely to be representative for all PWID living in Germany. E.g. our study sample might be more knowledgeable about their infection status than PWID living in smaller cities due to better access to drug user services, testing and treatment in larger cities.

## Conclusion

In our study, 17 % of HIV positive PWID and 27 % of those with chronic HCV infection were unaware of their infections. These results indicate that the majority of the study population is aware of their infection status, however still more than a quarter of those with infectious HCV and nearly one in five of HIV infected PWID in our study sample did not know their status, although they were often attached to opioid substitution therapy or other harm reduction services. Not being aware of the infection status implies that they cannot access appropriate health care and they risk unknowingly transmitting the disease to others.

In line with several other studies, we also believe that the quality of post-test counselling is crucial for increasing awareness of infection status as well as for ensuring a positive impact on risk behaviours and ensuring linkage to care and appropriate medical services for both infected and uninfected PWID. In the era of highly effective antiviral HCV-treatment options, the opportunity is there to clear infection in almost all HCV-infected PWID, if infected persons become aware of their status and are linked to care.

## Abbreviations

AB, antibodies; DBS, dried blood spots; HCV, Hepatitis C infection; MSM, men who have sex with men; PWID, people who inject drugs; RKI, Robert Koch Institute; UAM, unlinked anonymous monitoring
